# The transcriptional landscape of cancer stem-like cell functionality in breast cancer

**DOI:** 10.1186/s12967-024-05281-w

**Published:** 2024-06-03

**Authors:** Oana Baldasici, Olga Soritau, Andrei Roman, Carmen Lisencu, Simona Visan, Laura Maja, Bogdan Pop, Bogdan Fetica, Andrei Cismaru, Laurian Vlase, Loredana Balacescu, Ovidiu Balacescu, Aman Russom, Oana Tudoran

**Affiliations:** 1 Department of Genetics, Genomics and Experimental Pathology, The Oncology Institute “Prof. Dr. Ion Chiricuță”, Cluj-Napoca, Romania; 2 Department of Pharmaceutical Technology and Biopharmaceutics, University of Medicine and Pharmacy “Iuliu Hatieganu” , Cluj-Napoca, Romania; 3 Department of Radiology, The Oncology Institute “Prof. Dr. Ion Chiricuță”, Cluj-Napoca, Romania; 4 Department of Pathology, The Oncology Institute “Prof. Dr. Ion Chiricuță”, Cluj-Napoca, Romania; 5 Research Center for Functional Genomics, Biomedicine and Translational Medicine, University of Medicine and Pharmacy “Iuliu Hatieganu”, Cluj-Napoca, Romania; 6 Division of Nanobiotechnology, Department of Protein Science, KTH Royal Institute of Technology, Stockholm, Sweden

## Abstract

**Background:**

Cancer stem-like cells (CSCs) have been extensively researched as the primary drivers of therapy resistance and tumor relapse in patients with breast cancer. However, due to lack of specific molecular markers, increased phenotypic plasticity and no clear clinicopathological features, the assessment of CSCs presence and functionality in solid tumors is challenging. While several potential markers, such as CD24/CD44, have been proposed, the extent to which they truly represent the stem cell potential of tumors or merely provide static snapshots is still a subject of controversy. Recent studies have highlighted the crucial role of the tumor microenvironment (TME) in influencing the CSC phenotype in breast cancer. The interplay between the tumor and TME induces significant changes in the cancer cell phenotype, leading to the acquisition of CSC characteristics, therapeutic resistance, and metastatic spread. Simultaneously, CSCs actively shape their microenvironment by evading immune surveillance and attracting stromal cells that support tumor progression.

**Methods:**

In this study, we associated in vitro mammosphere formation assays with bulk tumor microarray profiling and deconvolution algorithms to map CSC functionality and the microenvironmental landscape in a large cohort of 125 breast tumors.

**Results:**

We found that the TME score was a significant factor associated with CSC functionality. CSC-rich tumors were characterized by an immune-suppressed TME, while tumors devoid of CSC potential exhibited high immune infiltration and activation of pathways involved in the immune response. Gene expression analysis revealed IFNG, CXCR5, CD40LG, TBX21 and IL2RG to be associated with the CSC phenotype and also displayed prognostic value for patients with breast cancer.

**Conclusion:**

These results suggest that the characterization of CSCs content and functionality in tumors can be used as an attractive strategy to fine-tune treatments and guide clinical decisions to improve patients therapy response.

## Introduction

Inside breast tumors, a complex and dynamic environment comprising heterogeneous populations of cancer cells, stromal cells, and immune infiltrates drives tumor growth, promotes metastatic spread [1], and hinders patients’ therapeutic response [2]. Among these cells, breast cancer stem-like cells (CSCs) [3], a population of slowly proliferating cells that exist within the tumor bulk and are usually quiescent in the G0 phase for long periods of time, are increasingly believed to drive breast tumor heterogeneity [4], promote metastasis [5], evade immune surveillance [6] and augment resistance to standard radiation and chemotherapy [7]. Current anticancer therapies target either the proliferation of tumor cells or the immune response against tumors; hence, quiescent CSCs may escape conventional treatments. CSCs are also preferentially selected during current therapies, and under favorable microenvironmental signals and cellular interactions within their niche, they can self-renew, driving tumor progression and metastasis [8].

CSCs have been isolated from human breast tumor samples and cancer cell lines mainly based on the expression of surface markers (e.g., CD44, CD24, and CD133) (rev in [9]). Several studies have shown that CD44+/CD24- cells increase therapeutic resistance [10] and invasiveness [11] and are correlated with a poor prognosis in breast cancer patients [12]. However, emerging evidence suggests that cancer cells exhibit high plasticity and that even cells that initially express a CD44^low^ phenotype might be able to convert into CD44^high^ CSCs in vivo [13]. Thus, culture adaptation and long-term subculture of individual CSC populations may induce genetic and phenotypic alterations in isolated cells that may not completely reflect all the biological features of primary CSCs [14]. Moreover, recent work has shown that CSCs are dynamically regulated by niche and microenvironmental cues, upon which they also exert regulatory effects [13, 15, 16]. Thus, instead of looking solely at the marker expression in these populations, the critical next steps should include understanding the factors that drive the CSC state and the emerging role of the surrounding multicellular architecture in determining CSC characteristics. There is a limited understanding of the degree to which CSC models can accurately recapitulate the distribution and functionality of CSCs observed in patients, and even though they are difficult to obtain, patient samples remain the gold standard for CSC investigations.

In this study, we explored the presence, functionality and microenvironmental landscape of CSCs in breast tumors from patients. We integrated in vitro mammosphere formation assays with transcriptomic profiling and deconvolution methods. We used matched breast tissues to perform both high-resolution transcriptional profiling via microarray analysis [17] and to generate matched CSC cultures [18]. This approach enabled a structured dissection of the CSC phenotype versus the TME-induced contribution to self-renewal and proliferation under anchorage-independent conditions. We calculated that CSC states are associated with distinct TMEs, suggesting that TME signals are critical regulators of the CSC state, plasticity, and response to therapy.

## Materials and methods

### Patients and biopsy samples collection

A total of 125 female patients diagnosed with invasive breast cancer at The Oncology Institute “Ion Chiricuță”, Cluj-Napoca, Romania (IOCN), were included in this study. American Joint Committee on Cancer (AJCC) TNM system was used for both clinical and histopathological staging of the patients. The study was approved by the IOCN ethical committee (Approval No. 59/29.11.2016), and all patients provided written consent for participation in the study in accordance with the Declaration of Helsinki. Two or three fresh breast tissue biopsies were collected via core needle aspiration under ultrasound guidance before patients received any treatment. The first biopsy samples were sent for pathologic analysis, while the second biopsy samples were used for mammosphere assays. Whenever possible, third biopsies were collected in RNAlater (Invitrogen, Waltham, MA, USA) and stored in liquid nitrogen for transcriptomic profiling.

### In vitro mammosphere formation assay

Biopsies were disaggregated mechanically into fragments of approximately 1 mm3 and digested enzymatically for 20 min at 37 °C in a collagenase solution to maximize the retrieval of single cells. After filtration through 70 μm Filcon filters, single-cell suspensions were cultured under anchorage-independent conditions according to the methods of Dontu et al. [18], with modifications. Mammospheres were grown in Ultra-Low Attachment plates (Corning, Corning, NY, USA) in specific growth media supplemented with serum-free RPMI w/o phenol red (Thermo Scientific, Waltham, MA, USA), 100 units/mL penicillin, 100 µg/mL streptomycin (Lonza, Basel, Switzerland), 2 mM glutamine (Lonza, Basel, Switzerland), 20 ng/mL basic fibroblast growth factor (b-FGF) (R&D Systems, Minneapolis, MN, USA), 10 ng/mL epidermal growth factor (EGF) (Sigma‒Aldrich, Saint Louis, MO, USA), 1X B27 and 1X N2 (Gibco, Thermo Scientific, Waltham, MA, USA) and incubated in a 37 °C incubator containing 5% CO2. Mammosphere formation was monitored by phase-contrast microscopy for 7 to 30 days using a Zeiss Zen inverted microscope with an attached Nikon camera. Spheroids size was assessed using the integrated measurement tool of the microscope-camera software (Zeiss-Zen).

### RNA extraction

Frozen biopsies were homogenized in TRIzol Reagent (Ambion, Life Technologies, Carlsbad, CA, USA) using a Miccra D-1 (Miccra GmbH, Mullheim, Germany) polytron and processed for total RNA extraction according to the manufacturer’s instructions. The total RNA concentration was analyzed with a NanoDrop ND-1000 (Thermo Scientific, Waltham, MA, USA), and the quality was assessed with a 2100 Bioanalyzer (Agilent Technologies, Santa Clara, CA, USA).

### Microarray analysis

Twenty-four biopsy samples were subjected to phenotype-associated transcriptomic analysis via microarray analysis. Biopsy samples were selected based on RNA quality. Microarray probes labeled with Cy3 were synthesized from 100 ng of total RNA with a Low Input Quick Amp Labeling Kit (Agilent Technologies, Santa Clara, CA, USA), and the quality and quantity were checked with a NanoDrop ND-1000 (Thermo Fischer Scientific, Wilmington, DE, USA). The probes were hybridized for 17 h at 65 °C on Agilent SurePrint G3 custom GE 4 × 180k arrays (Agilent Technologies, Santa Clara, CA, USA), which contained 100,744 unique sequences corresponding to mRNAs and lncRNAs. The slides were scanned with an Agilent G2505C Microarray Scanner (Agilent Technologies, Santa Clara, CA, USA) at 3 μm resolution, and the microarray images were processed with Agilent Feature Extraction software v. 11.5.1.1 (Agilent Technologies, Santa Clara, CA, USA).

### Bioinformatic analysis

Bioinformatic analysis was performed in R/Bioconductor using the raw median signals as input. Control and flagged spots were systematically removed. The data were quantile normalized between arrays, and the median signal value for the replicate probes on each array was computed. The differential expression was assessed using linear models and empirical Bayes statistics implemented in the limma package/R. The Bayesian model smoothens the standard errors of log-fold changes across genes, squeezing the gene-specific variances towards a common value derived from the entire dataset [19]. A minimum 1.5-fold decrease or increase in gene expression between groups with different mammosphere-forming capacities and a p value less than 0.05 were considered significant. Ingenuity Pathway Analysis software (IPA, Qiagen, Hilden, Germany) was used to map the differentially expressed genes (-1.5 ≥ FR ≥ 1.5) between the mammosphere formation groups into canonical pathways and biological functions as well as to visualize the upstream regulators of each pathway. An enrichment score [Fisher’s exact test (FET) P value] measuring the overlap of the observed and predicted regulated gene sets and a Z score assessing the match between the observed and predicted up- and downregulation patterns were automatically calculated by IPA algorithms [20]. The two scores were considered to indicate the most significant pathways (*p* < 0.05, -2 ≥ Z score ≥ 2) that were differentially regulated between groups. The abundance of specific cell types in breast cancer biopsies was estimated based on individual gene expression profiles by using the xCell deconvolution algorithm developed by Aran et al. [21]. This webtool (https://comphealth.ucsf.edu/app/xcell) uses gene expression signature to infer the abundance of immune and stromal cell types within bulk transcriptomics data, with the highest accuracy.

### Gene expression validation by qRT‒PCR

Biopsy samples collected from 60 breast cancer patients were used for microarray gene expression validation. Total RNA (500 ng) was reverse transcribed using a Transcriptor First Strand cDNA Synthesis Kit (Roche, Basel, Switzerland) following the random hexamer primer protocol. A total of 2.5 µl of 1:10 (v/v) diluted cDNA was amplified with a Light Cycler TaqMan Master Kit (Roche, Basel, Switzerland) in a final volume of 10 µl using a LightCycler 480 II Thermocycler (Roche, Basel, Switzerland). PCR was performed according to the following program: activation step at 95 °C for 10 min; followed by amplification step of 40 cycles: denaturation at 95 °C for 10 s, annealing at 55 °C for 20 s, and extension at 72 °C for 1 s; ending with a cooling step at 40 °C for 30 s. The relative expression levels of the target genes were quantified using the ΔΔCt method after normalization to the 18 S housekeeping gene.

### Clinical and TCGA data analysis and integration

The mRNA expression of breast invasive carcinoma (TCGA, PanCancer Atlas) and clinical information were obtained from the cBioPortal for Cancer Genomics (https://www.cbioportal.org/). For clinical data associations, gene expression was analyzed and plotted in relation to clinical parameters, while for survival curves, patients were divided into high versus low-expression groups based on the median value of each gene.

### Immunohistochemical staining

Biopsy specimens obtained in the pathology department were fixed with 10% neutral buffered formalin for 6–72 h at room temperature and routinely processed. The paraffin blocks were sectioned at 4 μm, and the slides were immersed for five minutes in xylene for deparaffinization and twice in histology-grade alcohol for one minute for dehydration. Manual and automated IHC were used. The manual IHC protocol used the heat-induced epitope retrieval (HIER) method for antigen retrieval and solutions based on citrate, with a pH = 6 for most antibodies or a pH = 9 for CD15 and SMA. The antibodies used were CD4 (Novocastra, Leica Biosystems, Wetzlar, Germany), clone [4B12], dilution 1:50, CD1a (Dako, Agilent Technologies, Santa Clara, CA, USA)-clone [010], dilution 1:50, smooth muscle actin (SMA) (Dako, Agilent Technologies, Santa Clara, CA, USA) clone [1A4], dilution 1:100, 2D7- anti-Basophil antibody (Abcam, Cambridge, UK) clone [2D7], dilution 1:50, ready to use CD15 (Dako, Agilent Technologies, Santa Clara, CA, USA), clone [Carb-3], CD1c (Abcam, Cambridge, UK), clone [OTI2F4], dilution 1:350, CD66b (BioLegend, San Diego, CA, USA), clone [G10F5], 1:50, Langerin (Novocastra, Leica Biosystems, Wetzlar, Germany), and clone [12D6], dilution 1:50. The mixture was incubated for 30 min at 37 °C. A Novolink Polymer Detection System (Novocastra, Leica Biosystems, Wetzlar, Germany) was used for visualization. CD8 staining was performed by automated IHC using a Ventana BenchMark Ultra and an SP57 clone (ready to use). Automated IHC staining was performed using a Ventana OptiView Dab (Ventana Medical Systems, Oro Valley, AZ, USA) detection kit. Following the visualization step, the slides were coverslipped and analyzed. Slides were reviewed by a pathologist. The evaluation protocol included slide review and selection of the area with the highest IHC expression. A semiquantitative method was used to assess the percentage of cells in the peritumoral stroma that expressed the IHC markers. The assessment was performed in the area with the highest expression.

### Statistical analysis

Correlations between mammosphere formation and clinicopathological data or neo-adjuvant therapy response were analyzed using Fisher’s exact test in SPSS (IBM SPSS Statistics for Macintosh, Version 28.0; IBM Corp Armonk, NY, USA). The rest of statistical analyses and graph plotting were carried out in GraphPad Prism (GraphPad Software, Version 8 for Windows; Boston, Massachusetts USA). Chi-squared test was used to analyze the correlation between cellular subsets identified by deconvolution algorithm and mammosphere formation groups. Spearman’s rank correlation coefficient was used to test the relationship between the infiltration patterns of immune cell subsets with each other and with MSCs. The associations between tissue mRNAs expression and mammosphere formation status or clinicopathological characteristics were evaluated with Mann-Whitney U test for two categorical variables or Kruskall-Wallis test, followed by Dunn’s multiple comparison post hoc test, in case of three or more categorical variables. In order to evaluate the association between progression-free survival (PFS) and mRNAs of interest, each mRNA expression was classified according to its median value, based on which the patients were allocated into high- or low-expression group. The survival curves were estimated using the Kaplan-Meier method, and log-rank test was used to compare the survival distributions. All analyses were considered significant at p-value less than 0.05. The heatmap and clustering of IFNγ activated genes was generated using the Morpheus online tool (https://software.broadinstitute.org/morpheus/).

### Data availability

The datasets supporting the conclusions of this article are available in the Gene Expression Omnibus (GEO) at GSE244973 (https://www.ncbi.nlm.nih.gov/geo/query/acc.cgi?acc=GSE244973). The expression profile data analyzed in this study were obtained from the cBioPortal for Cancer Genomics (TCGA BRCA database).

## Results

### The stem-like potential of breast cancer biopsies

**Fig. 1:**
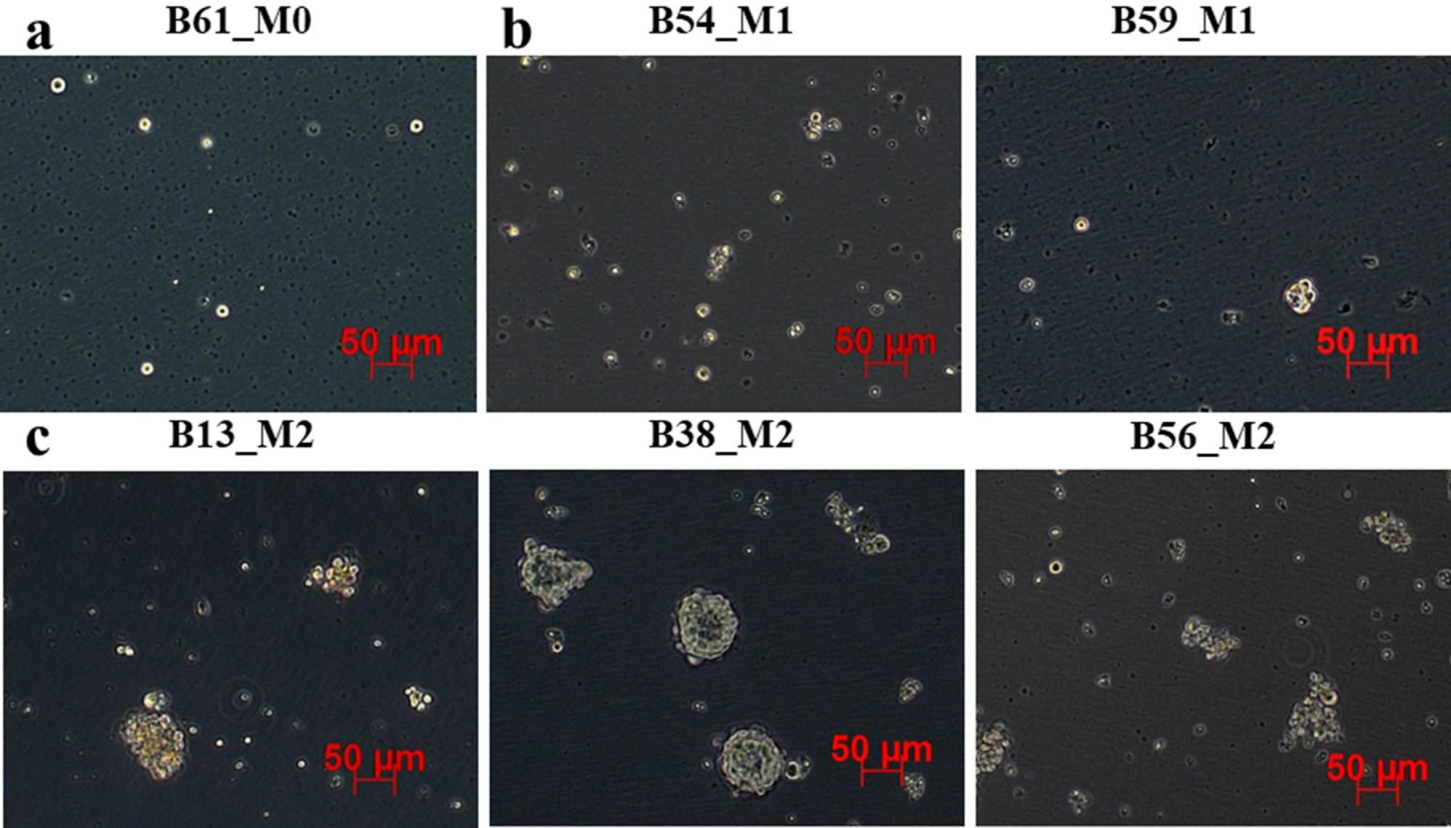
Mammosphere cultures from breast cancer biopsies. **a** M0 cultures lacking cells with stem potential. **b** M1 cultures containing mostly single cells and quiescent clusters (< 50 μm). **c** M2 cultures with self-renewing cells in mammospheres (> 50 μm)

A cohort of 125 biopsies from breast cancer patients was evaluated for CSC functionality via mammosphere assay. Visual observation of the mammosphere cultures and spheroids measurements revealed 3 main phenotypes. Some biopsies formed cultures with large spheres (more than 50 μm in diameter, previously reported as mammospheres with stem-like features [22]), some grew as small spheres (less than 50 μm in diameter) and single living cells that were viable but non-dividing even after one month of culturing, while other cultures presented no spheroids with mostly dead single cells and debris. Therefore, biopsies that produced cultures containing spheroids with a diameter larger than 50 μm were considered enriched in self-renewing CSCs. The intermediate phenotype of cultures presenting small spheroids and single living cells was attributed to the presence of quiescent or more differentiated CSCs, which had lower self-renewal abilities. Tumor samples that led to cultures without any viable cells were considered void of CSCs. Both the mammospheres and the small spheroids showed significant variability across samples. Some mammospheres grew as tight, round spheroids, whereas others formed rather loose and irregularly shaped conglomerates. For easier analysis and subsequent reference, biopsies enriched in proliferating CSCs were labeled with M2, biopsies with quiescent CSCs were labeled with M1, and biopsies devoid of CSCs were labeled with M0 (Fig. 1). Of all 125 biopsies, 24% were M2, 32% were M1 and 44% were M0.

Furthermore, we analyzed the relationship between mammosphere formation in vitro and the clinicopathological characteristics of the breast cancer patients included in the study (Table 1). Statistically significant associations (*p* = 0.019) were observed only between the mammosphere formation potential and clinical tumor size. Correlations between mammosphere formation and pathological response to neoadjuvant therapy were also investigated, but no statistically significant differences were detected (Table 2).

**Table 1:** Correlations between mammosphere formation and clinicopathological data

**Distribution by clinical characteristics**		**M0**	**M1**	**M2**	**Fisher’s exact test** **p value**
		***n*** ** = 55 (44%)**	***n*** ** = 40 (32%)**	***n*** ** = 30 (24%)**	
Age (Median = 61)	≤ 60 > 60	24 (19.2%) 31 (24.8%)	22 (17.6%) 18 (14.4%)	13 (10.4%) 17 (13.6%)	0.505
Grading (biopsy)	1	7 (5.6%)	8 (6.4%)	3 (2.4%)	0.805
	2	34 (27.2%)	22 (17.6%)	17 (13.6%)	
	3	14 (11.2%)	10 (8%)	9 (7.2%)	
	NA	0 (%)	0 (%)	1 (0.8%)	
ER	+	42 (33.6%)	31 (24.8%)	25 (20%)	0.818
	- (≤10%)	13 (10.4%)	9 (7.2%)	5 (4%)	
PR	+	31 (24.8%)	24 (19.2%)	19 (15.2%)	0.821
	- (≤20%)	24 (19.2%)	16 (12.8%)	11 (8.8%)	
Her2	+	3 (2.4%)	5 (4%)	3 (2.4%)	0.473
	-	50 (40%)	35 (28%)	24 (19.2%)	
	NA	2 (1.6%)	0 (0%)	3 (2.4%)	
Ki67	≤ 20%	26 (20.8%)	16 (12.8%)	14 (11.2%)	0.708
	> 20%	28 (22.4%)	24 (19.2%)	14 (11.2%)	
	NA	1 (0.8%)	(0%)	2 (1.6%)	
Molecular Subtype	Luminal A	16 (12.8%)	13 (10.4%)	10 (8%)	0.856
	Luminal B	24 (19.2%)	15 (12%)	11 (8.8%)	
	Her2+	3 (2.4%)	5 (4%)	3 (2.4%)	
	TNBC	10 (8%)	7 (5.6%)	3 (2.4%)	
	NA	2 (1.6%)	0 (0%)	3 (2.4%)	
Tumor size (clinic)	cT1	6 (4.8%)	5 (4%)	3 (2.4%)	0.019
	cT2	25 (20%)	13 (10.4%)	19 (15.2%)	
	cT3	6 (4.8%)	3 (2.4%)	4 (3.2%)	
	cT4	9 (7.2%)	15 (12%)	1 (0.8%)	
	NA	9 (7.2%)	4 (3.2%)	3 (2.4%)	
Lymph nodes (clinic)	cN0	11 (8.8%)	12 (9.6%)	10 (8%)	0.161
	cN1	20 (16%)	7 (5.6%)	7 (5.6%)	
	cN > 2	14 (11.2%)	17 (13.6%)	10 (8%)	
	NA	10 (8%)	4 (3.2%)	3 (2.4%)	
Metastasis (clinic)	cM0	39 (31.2%)	31 (24.8%)	27 (21.6%)	-
	cM1	2 (1.6%)	3 (2.4%)	0 (0%)	
	NA	14 (11.2%)	6 (4.8%)	3 (2.4%)	
Clinical stage	I	2 (1.6%)	5 (4%)	2 (1.6%)	0.121
	II	17 (13.6%)	9 (7.2%)	16 (12.8%)	
	III	22 (17.6%)	16 (12.8%)	9 (7.2%)	
	IV	4 (3.2%)	3 (2.4%)	0 (0%)	
	NA	10 (8%)	7 (5.6%)	3 (2.4%)	
Tumor size (pathologic)	pT0	3 (2.4%)	6 (4.8%)	4 (3.2%)	0.469
	pT1	21 (16.8%)	15 (12%)	10 (8%)	
	pT > 2	16 (12.8%)	9 (7.2%)	11 (8.8%)	
	NA	15 (12%)	10 (8%)	5 (4%)	
Lymph nodes (pathologic)	pN0	23 (18.4%)	15 (12%)	15 (12%)	0.782
	pN1	9 (7.2%)	6 (4.8%)	7 (5.6%)	
	pN > 2	10 (8%)	9 (7.2%)	4 (3.2%)	
	NA	13 (10.4%)	10 (8%)	4 (3.2%)	
Lymphatic invasion (pathologic)	L0	27 (21.6%)	22 (17.6%)	14 (11.2%)	0.325
	L1	15 (12%)	8 (6.4%)	12 (9.6%)	
	NA	13 (10.4%)	10 (8%)	4 (3.2%)	
Survival status	Alive	46 (36.8%)	35 (28%)	25 (20%)	0.855
	Deceased	9 (7.2%)	5 (4%)	5 (4%)	

ER- estrogen receptor; PR- progesterone receptor; Her2 – human epidermal growth factor receptor; Ki67- cellular marker for proliferation; TNBC – triple negative breast cancer; cT – clinical tumor size; cN- clinical number of lymph nodes; cM – clinical metastasis; pT – pathological tumor size; pN- pathological number of lymph nodes; L – pathological lymphatic invasion

**Table 2:** Correlations between mammosphere formation and neoadjuvant therapy response

**Neoadjuvant therapy**	M0	M1	M2	Fisher’s exact test p value
	**n = 35 (42.16%)**	**n = 26 (31.32%)**	n = 22 (26.5%)	
**Only CT**	21 (25.3%)	16 (19.28%)	10 (12.05%)	-
**Only HT**	6 (7.23%)	2 (2.41%)	8 (9.64%)	
**CT + HT**	4 (4.82%)	2 (2.41%)	2 (2.41%)	
**Combinatory**	1 (1.2%)	2 (2.41%)	0 (0%)	
**Her2 + TT**	3 (3.61%)	4 (4.82%)	2 (2.41%)	
**Pathological Response**				
**Miller Payne**				
**Grade 1**	8 (9.64%)	4 (4.82%)	9 (10.84%)	0.286
**Grade 2**	4 (4.82%)	3 (3.61%)	1 (1.2%)	
**Grade 3**	8 (9.64%)	7 (8.43%)	6 (7.23%)	
**Grade 4**	7 (8.43%)	1 (1.2%)	1 (1.2%)	
**Grade 5**	4 (4.82%)	7 (8.43%)	4 (4.82%)	
**NA**	4 (4.82%)	4 (4.82%)	1 (1.2%)	
**RCB**				
**RCB-0**	3 (3.61%)	5 (6.02%)	4 (4.82%)	0.692
**RCB-I**	5 (6.02%)	2 (2.41%)	1 (1.2%)	
**RCB-II**	16 (19.28%)	9 (10.84%)	12 (14.46%)	
**RCB-III**	7 (8.43%)	6 (7.23%)	4 (4.82%)	
**NA**	4 (4.82%)	4 (4.82%)	1 (1.2%)	

CT- chemotherapy, HT- hormonal therapy, TT-targeted therapy, RCB- residual cancer burden, NA- data not available

### Molecular profiling of breast cancer biopsies

**Fig. 2:**
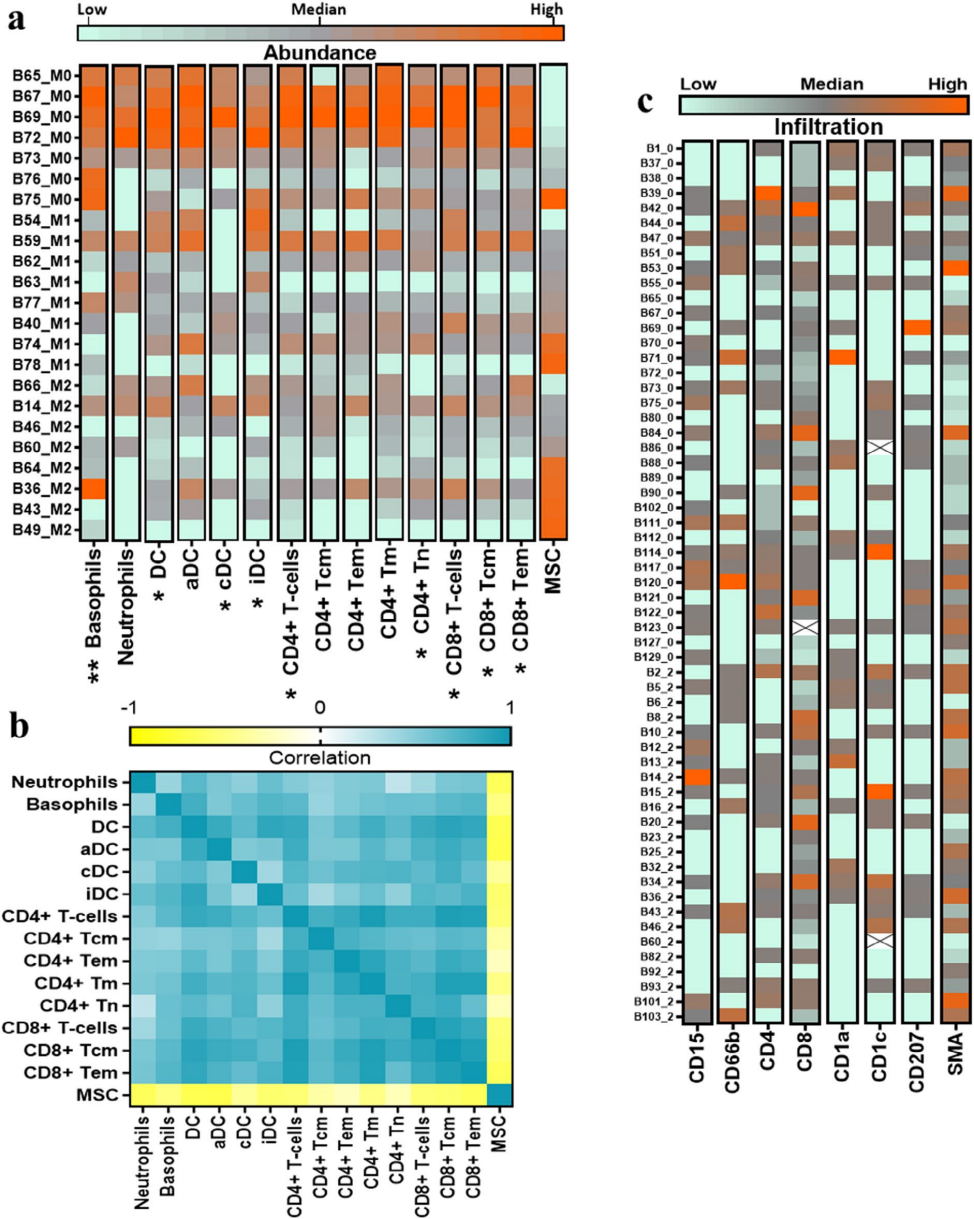
Microenvironment landscape of M0, M1 and M2 biopsies. **a** Heatmap displaying the abundance of microenvironment cells (DC-dendritic cells; aDC-activated dendritic cells; cDC- conventional dendritic cells; iDC-immature dedritic cells; CD4 + Tcm- CD4 + central memory T cells; CD4 + Tem- CD4 + effector memory T cells; CD4 + Tm- memory CD4 + cells; CD4 + Tn – naïve T cells; CD8 + Tcm- CD8 + central memory T cells; CD8 + Tem- CD8 + effector memory T cells; MSC – mesenchymal stem cells; ) in each tumor biopsy; * indicates the cell types with infiltrating levels significantly associated (chi-square test, * *p* < 0.05, ** *p* < 0.01) with the mammosphere-forming potential of breast biopsies. **b** Association between infiltrated immune cells and MSC abundance in tumor biopsies (Spearman test). **c** Immunohistochemical evaluation of immune and stromal cells within breast cancer biopsies

Whole-genome transcriptomic analysis was performed on 24 selected samples, 8 from each mammosphere formation group. Samples were selected based on availability at the time of analysis and an RNA Integrity Number (RIN) > 8. A single M0 sample was excluded from the bioinformatic data analysis, a second examination by the pathologist confirmed it to be a metastasis of malignant melanoma. To ensure the integrity of the analysis and prevent potential technical biases stemming from variations in microarray runs, only the 23 samples assessed in the same microarray run were employed for subsequent analysis. Microarray data containing gene expression information for the remaining 23 biopsies were loaded into the xCell deconvolution webtool [21] and subjected to computational dissection to estimate the cellular composition of the tumor microenvironment. Bulk tumor deconvolution algorithm revealed a distinctive mixture of infiltrating immune cells and mesenchymal stem cells within each mammosphere formation group (Fig. 2a). Biopsies from the M0 group displayed the highest abundance scores for infiltrating innate and adaptive immune cells. M2 biopsies with actively proliferating stem-like cells exhibited a rather immune-depleted outlook and were enriched in cells presenting a mesenchymal stem cell (MSC) signature. M1 biopsies revealed an intermediate state of immune infiltration and lower scores for MSCs. Of the 15 different cellular subsets identified by computational deconvolution, the abundance scores of 9 cell types, namely, basophils (χ^2^= 9.47, *p* = 0.008), dendritic cells, and CD4 + and CD8 + T lymphocytes (χ^2^= 6.039, *p* < 0.05), were significantly associated with the mammosphere formation potential of the M0, M1 and M2 groups. (Fig. 2a, cell types displaying significant associations are marked with *).

Furthermore, the infiltration of immune cells displayed significant positive correlations with each other, suggesting potential cooperation. Several immune cell subsets presented opposite infiltration patterns to those of MSCs, with the strongest trends being observed for dendritic cells and activated dendritic cells (Spearman ρ=-0.68 and ρ=-0.70, *p* < 0.001), neutrophils (Spearman ρ=-0.65, *p* < 0.001) and CD8 + T effector memory cells (Spearman ρ=-0.65, *p* < 0.001) (Fig. 2b). Significant inverse correlations (*p* < 0.03) were also detected between the abundance of MSCs and immature dendritic cells, basophils, CD4 + T cells, CD4 + memory T cells, CD8 + T cells and CD8 + central memory T cells (Spearman ρ= -0.57, -0.5, -0.55, -0.48, -0.54 and − 0.57, respectively) (Fig. 2b).

Next, using immunohistochemical staining, we explored the abundance of the significantly associated immune and stromal cells within matching formalin-fixed, paraffin-embedded (FFPE) tissues collected for diagnosis. CD8 and CD4 surface markers were used to stain for CD8 + and CD4 + T cells; CD1a, CD1c, and langerin (CD207) were used for dendritic cells; 2D7, for basophils; CD15 and CD66b, for neutrophils; and smooth muscle actin (SMA), for fibroblasts and/or mesenchymal stem cells. The percentage of positive cells in each biopsy was characterized as high or low according to the median value (Fig. 2c). Significant associations were found only between the M2 subgroup and the upper quartile of SMA-expressing cells (chi-square test, χ^2^ = 7.693, *p* = 0.02).

**Fig. 3:**
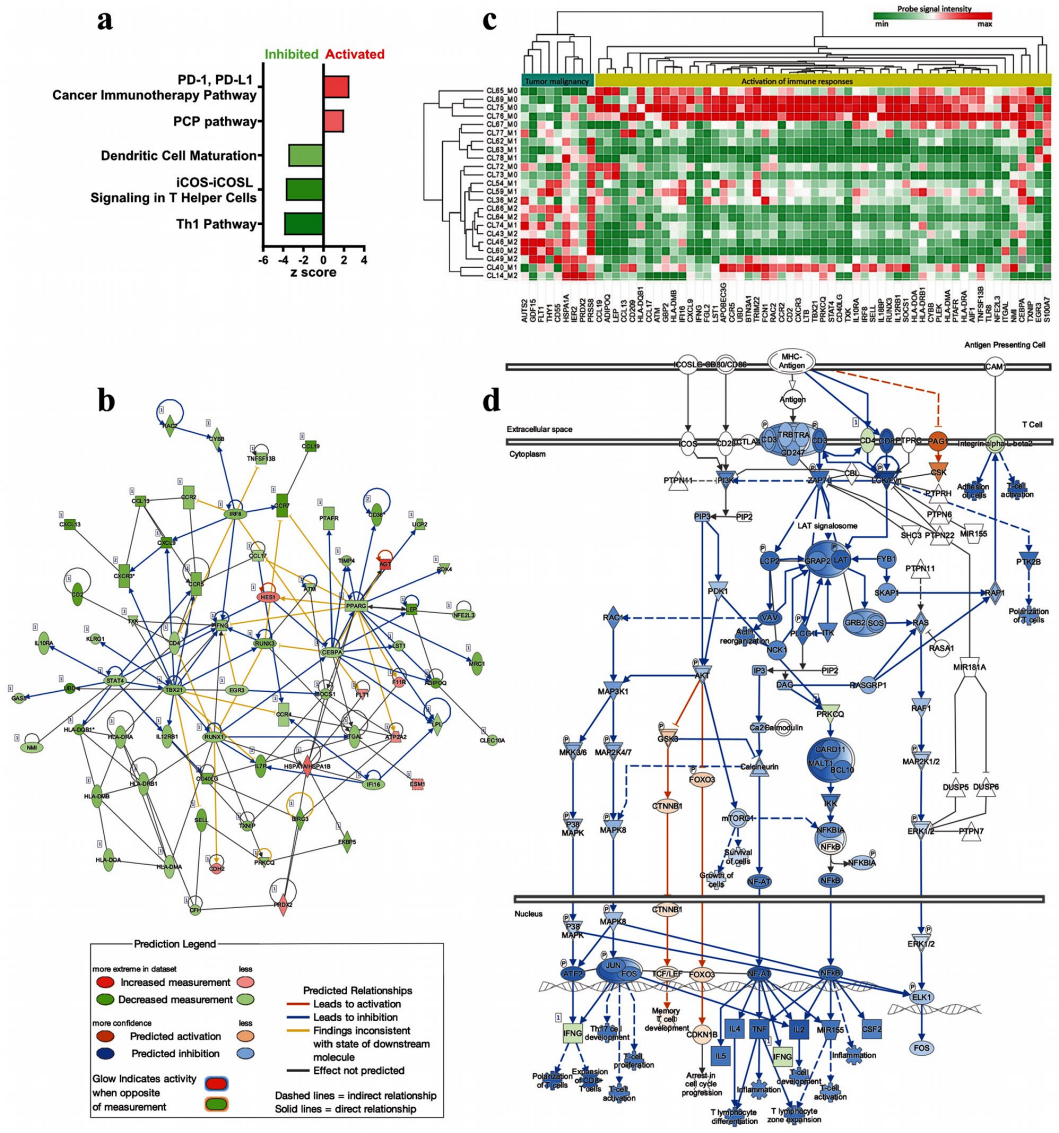
**a** Functional integration of bulk transcriptomic signatures revealed divergent activation of immunological and stemness-related signaling pathways between the M2 and M0 mammosphere-forming groups. **b** Network analysis of direct interaction between IFNG and target genes in the M2 vs. M0 dataset; **c** Expression of downstream genes consistent with IFNG inhibition in M0, M1, and M2 tumors and related biological processes; **d** Predicted regulation of the T cell receptor downstream signaling pathway in M2 vs. M0 dataset

To further explore the relevant regulators that drive the observed differences in mammosphere formation, we performed differential expression analysis of the transcriptomic signatures between the three groups of tumors. A threshold of -1.5 ≥ FR ≥ 1.5 and p value < 0.05 was considered for filtering significantly differentially expressed genes (DEG). A total of 908 genes were found to be differentially expressed between M2 and M0, 658 between M1 and M0 and 395 between M2 and M1, before applying false discovery rate correction. Adjusting p-values by Benjamini–Hochberg method, further filtered the gene set leading to loss of statistical significance. These results are expected when stringent corrections are employed for groups that differ minimally. Thus, considering the distinct phenotypes used to separate the groups, we decided to further explore the possible underlying factors and retained only the FR (fold regulation) and unadjusted p values (-1.5 ≥ FR ≥ 1.5, p value < 0.05) as the significance thresholds. The gene sets were uploaded to IPA for functional analysis. When comparing the activation levels of several signaling pathways, the most pronounced differences were detected between the M2 and M0 groups, with M1 exhibiting an intermediate phenotype. The analysis revealed the inhibition of several pathways associated with lymphocyte activation (ICOS-ICOSL signaling pathway in T helper cells, Z score = -3.74, *p* < 0.001) and differentiation (Th1 pathway, Z score = -3.9, *p* < 0.001) as well as dendritic cell maturation (Z score = -3.5, *p* < 0.001) in the M2 mammosphere-forming group compared with the M0 group. Additionally, the analysis revealed two significantly activated pathways (*p* < 0.05): the PD-1, PD-L1 cancer immunotherapy pathway (Z score = 2.53, *p* < 0.001), and the PCP pathway (Z score = 2, *p* < 0.05) in M2 biopsies compared with M0 biopsies (Fig. 3a). Taken together, these results suggest a reduced immune response in tumors bearing CSCs.

Upstream regulator analysis highlighted IFNG as an important upstream regulator of the significant signaling pathways and biological processes between the two groups. Network analysis of known direct interactions between IFGN and its target genes revealed the predicted mechanistic orientation between the upstream regulator and the target genes in our dataset (Fig. 3b**)**. Therefore, we explored the IFNγ downstream signaling pathways in each mammosphere-forming group by mapping the target genes consistent with IFNγ activation to a heatmap representing the transcript abundance of each gene. This representation revealed individual activation patterns of IFNγ-stimulated genes in the M0, M1 and M2 groups.

The upregulated genes consistent with IFNγ signaling activation were predicted by IPA to be involved in solid tumor malignancy, while the downregulated genes consistent with the inhibition of IFNγ signaling were predicted to be involved in cellular movement, lymphocyte, and leukocyte migration and blood cell activation. Additionally, unsupervised hierarchical clustering revealed a clear separation between the M0 and M2 mammosphere-forming groups in terms of IFNγ signaling, with greater activation in the M0 group than in the M2 group (Fig. 3c). Thus, we hypothesize that impairment of IFNγ signaling results in the suppression of T cell mediated immunity (Fig. 3d) and CSC maintenance in breast tumors.

**Fig. 4:**
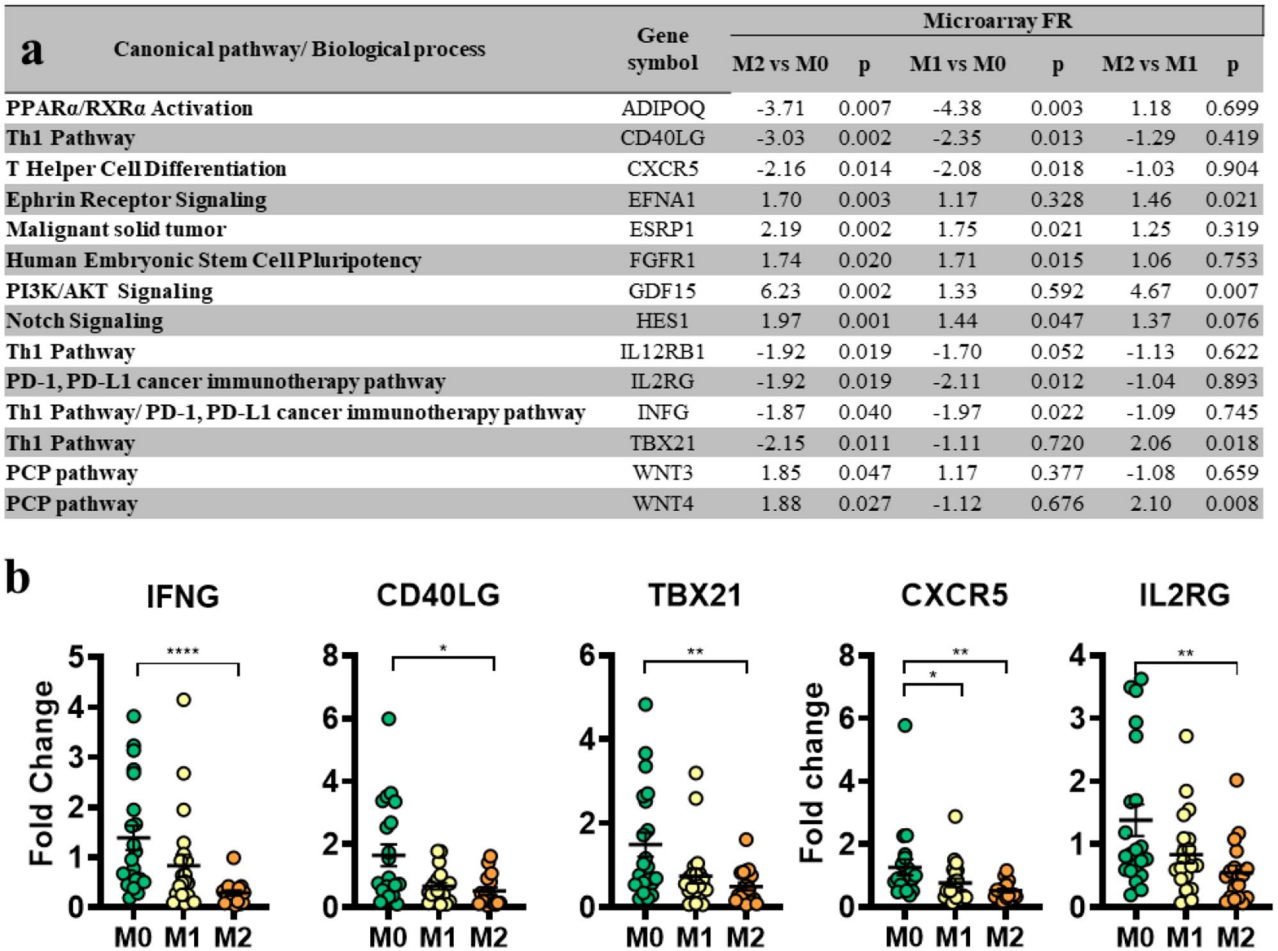
Gene expression validation in an extended cohort of patient samples **a** The 14-gene set selected from pathways related to stem cell signaling and immune activation. **b** qRT‒PCR validation of differentially expressed genes in M0, M1 and M2 biopsies from 60 patients

A set of 14 genes (-1.5 ≥ FR ≥ 1.5, *p* < 0.05 in M2 vs. M0), which are involved in different stem cell-related and immune activation signaling pathways (Fig. 4a), were considered for further gene expression validation via qRT‒PCR. The validation cohort consisted of 60 patient biopsies, comprising the initial 23 samples used for microarray analysis and 37 additional samples from the biobank. Sample selection was based on RNA quality and included tissue specimens from all three mammosphere-forming groups. The expression levels of ADIPOQ, CD40LG, CXCR5, EFNA1, ESRP1, FGFR1, GDF15, HES1, IL12RB1, IL2RG, INFG, TBX21, WNT3, and WNT4 were evaluated in the mammosphere-forming groups via qRT‒PCR. Of these, IFNG, CD40LG, TBX21, and IL2RG were significantly downregulated in the M2 group compared to the M0 group. CXCR5 was significantly downregulated in both the M2 and M1 groups compared to the M0 group (Fig. 4b).

**Fig. 5:**
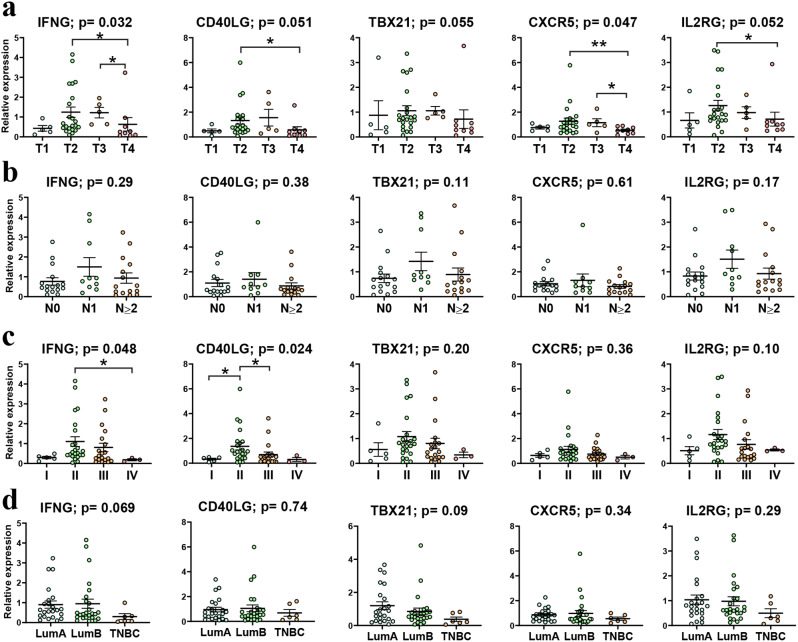
Associations between gene expression and clinicopathological data of breast cancer patients: **(a)** tumor size, **(b)** lymph node metastasis, **(c)** clinical stage, and **(d)** molecular subtype; each graph depicts the p value of Kruskal-Wallis test, and * p-value < 0.05, **p-value < 0.01 were added where Dunn’s multiple-comparison test returned significant values

Next, we explored the associations between gene expression and clinicopathological data of our cohort of patients (Fig. 5). IFNG and CXCR5 were significantly associated with tumor size (*p* = 0.032 and *p* = 0.047, respectively), and their expression was lower in invading tumors (T4). Additionally, IFNG and CD40LG were associated with clinical stage (Kruskal-Wallis Test: *p* = 0.048 and 0.024, respectively), with relatively lower expression levels in more advanced stages (stage IV vs. stage II, for INFG; stage III vs. II for CD40LG). No statistically significant associations were found between TBX21 and clinical data in our cohort. Expression levels of CD40LG and IL2RG presented borderline significant association values with tumor size.

**Fig. 6:**
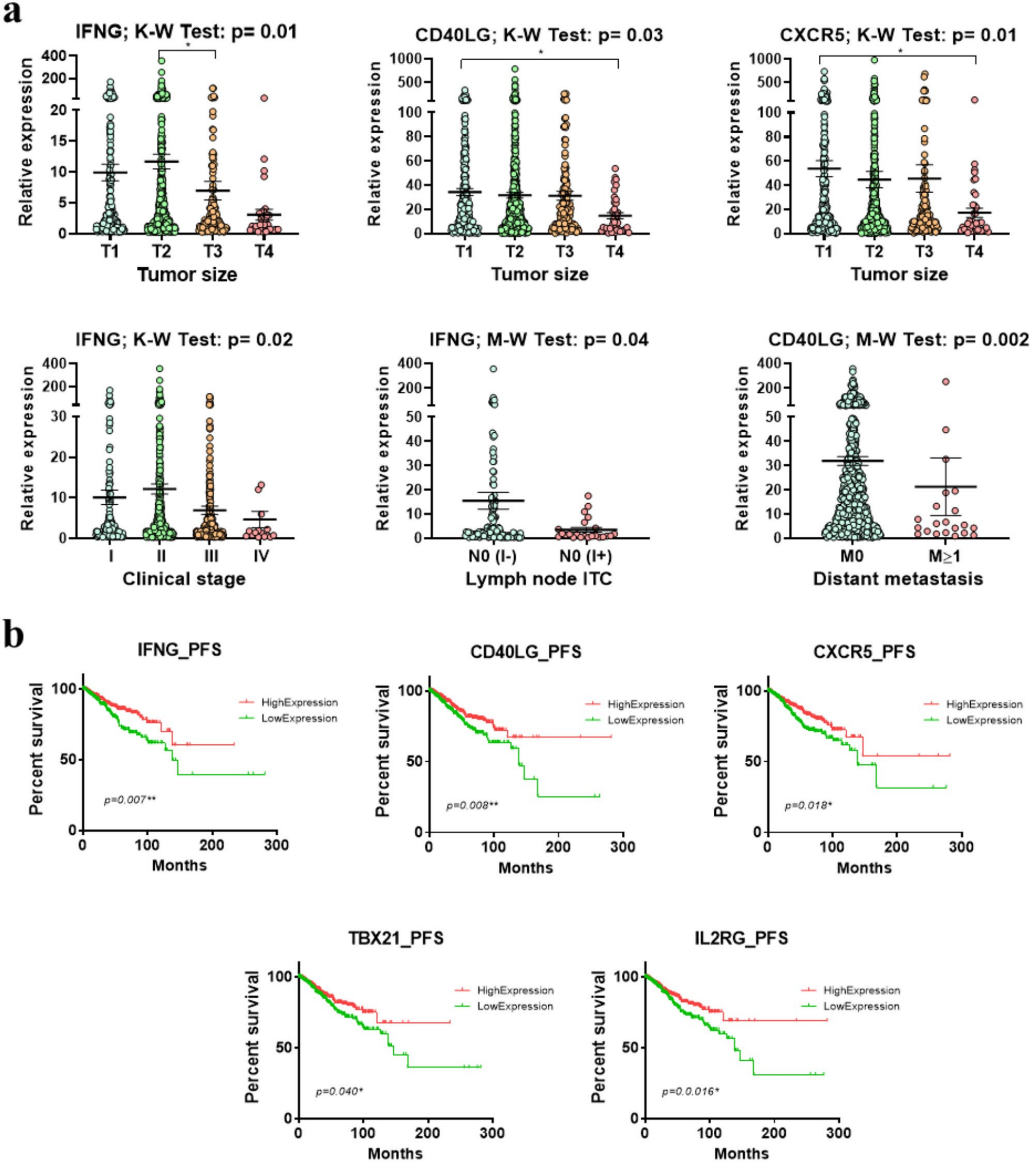
Relationships between immune response genes associated with mammosphere formation and clinical data from large cohorts of patients from TCGA. **a** Associations between the relative expression of genes involved in immune responses and clinical data of breast cancer patients from the TCGA database **b** Relationships between validated genes and PFS in breast cancer patients from TCGA

Furthermore, we used the TCGA BRCA dataset to evaluate the clinical significance of the validated genes in a larger patient cohort (Fig. 6a). Consistently, we found significant associations between lower expression of IFNG, CD40LG, and CXCR5 and larger tumor sizes or adjacent tissue invasion (*p* = 0.01, *p* = 0.03 and *p* = 0.01, respectively). Underexpressed IFNG was also associated with more advanced disease (*p* = 0.02) and the presence of isolated tumor cells within the lymph nodes (N0 I+, *p* = 0.04). Moreover, low CD40LG expression was associated with distant metastasis (*p* = 0.002). K‒M analysis (Fig. 6b) revealed that patients with higher expression levels of all the tested genes had significantly better progression-free survival (PFS). Moreover, higher IFNG expression was also associated with disease-free survival (*p* = 0.038), disease-specific survival (*p* = 0.027) and overall survival (*p* = 0.001) before 200 months (*p* = 0.027). Additionally, the high-expression CD40LG patient group had better disease-specific survival before 200 months (*p* = 0.038) than did the low-expression group.

## Discussion

CSCs have been previously analyzed in several solid tumor types [22–28], including breast cancer, but most of these studies have been performed on cell lines. The differences between primary and immortalized cells are stirring controversies regarding prospective characterization markers. For example, combinations of different markers, such as CD133, CD44, CD24, and ALDH, have been reported to be associated with cancer stem cell properties [9]; however, highly sensitive and specific markers for CSCs remain elusive. Moreover, marker-based detection of CSCs can only reveal the expression of certain molecules in a single snapshot in time, without considering the dynamic behavior of cancer cells or their ability to regulate signaling patterns in response to external cues [10]. Furthermore, this approach neglects the selection pressure of different microenvironments, excluding the possibility of spontaneous conversion of differentiated tumor cells to a stem-like state [15]. Thus, cell selection based on single or multiple surface expression markers could lead to misclassification of cell populations with stem properties. Grimshaw et al. [22] demonstrated that the mammosphere assay is a highly appropriate model for isolating and enriching tumorigenic stem-like cells from breast cancer patient samples and that the ability of cells to form mammospheres was not correlated with the CD44^+^/CD24^low/−^ phenotype.

Thus, combining the mammosphere assay with the use of primary cells obtained from biopsy samples is considered to be more relevant for analyzing the stemness properties of individual tumors, in order to develop more personalized therapeutic strategies. Additionally, one main advantage of using primary cell cultures over cancer cell lines is the availability of clinicopathological data from patients, which can be correlated with data obtained in vitro. To date, few studies [29, 30] have reported primary CSC cultures from breast cancer patients, and given the difficulties in establishing these primary cultures, these studies rely on small cohorts of patients.

Therefore, we used a mammosphere formation assay on patient-derived primary cells as a model to assess the presence and functionality of unsorted cancer stem-like cell populations in a cohort of 125 breast tumors. Our results revealed the heterogeneity of breast cancer tumors in terms of CSC content and functionality, generating three groups of primary cultures: highly enriched in mammospheres (M2 group), poorly enriched in small clusters and single living cells (M1), or devoid of living cells (M0). While the M0 group could be defined as a non-stem group, the M2 and M1 mammosphere-forming groups might exhibit two alternative states of cancer stem cell plasticity: an epithelial-like proliferative state (M2) and a mesenchymal-like, quiescent state (M1). Studies have shown that depending on microenvironmental cues, CSCs can adopt either state or can transition from a proliferative to a quiescent state and vice versa [5]. These findings suggest that breast tumor heterogeneity could be attributed to the presence of cancer cells with stem-like properties, which can proliferate and self-renew when seeded in the right environment [31, 32]. 

Within this context, our analysis aimed to investigate the tumor microenvironment (TME) characteristics associated with the maintenance of CSCs in breast tumors. By employing computational deconvolution techniques and pathway analysis of transcriptomic signatures derived from tumor biopsies, we calculated distinct patterns of immune infiltration in different groups based on mammosphere formation. We observed high levels of immune cell infiltration in the M0 group, intermediate levels in the M1 group, and low levels in the M2 group. Specifically, the M0 tumors exhibited a significantly greater abundance of lymphocytes, dendritic cells, and basophils than did the M2 tumors. Furthermore, we observed increased activation of immune response pathways in the M0 group, which correlated with the absence of mammosphere formation potential.

Conversely, the M2 biopsies showed a significant increase in the abundance of mesenchymal stem cells (MSCs) compared to that in the M0 group. Additionally, we observed activation of the planar cell polarity (PCP) developmental signaling pathway and the PD-1/PD-L1 immune evasion pathway in the M2 mammosphere group.

Based on these results, we hypothesize that the milieu-related factors specific to the TME present in each mammosphere formation group may drive CSC survival and induce a proliferative or quiescent state through autocrine (cancer cells themselves) and paracrine (surrounding stromal and immune cells) signaling feedback loops. Moreover, by upregulating immune evasion pathways, CSCs might block the infiltration and activity of immune effector cells within the tumor, attract stromal cells, and actively shape their niche [33]. Additionally, the stromal cell compartment plays an important role in tumor progression by driving the migration and invasion of tumor cells [34], increasing cancer cell proliferation [35], promoting in vitro tumorsphere formation, and promoting in vivo tumorigenesis [36].

Within the TME, secreted cytokines play a crucial role in regulating the activity of immune cells and their interaction with tumors. IFNγ is considered a major effector of immunity because it coordinates biological functions involved in host defense and the establishment of adaptive immunity and has critical roles in immune surveillance [37, 38]. In addition, IFNγ has a proapoptotic effect on cancer cells, limiting tumor growth in vivo [39].

Our data revealed IFNG (interferon gamma) as the upstream regulator of the pathways associated with the differential expression between the mammosphere formation groups. Additionally, the expression of the IFNG downstream genes displayed divergent activation patterns in the M0 and M2 groups, indicating impaired immune function coupled with the activation of malignancy-related pathways in the M2 biopsies. Therefore, we hypothesize that the immune evasion phenotype associated with the M2 phenotype might be driven by impaired IFNγ secretion and signaling within the TME.

Our hypothesis is supported by published data that demonstrate that in the context of chronic inflammation within the TME, cancer cells develop coping mechanisms designed to promote their immune evasion capabilities. Prolonged IFNγ exposure induces the overexpression of the inhibitory molecules PD-L1 and PD-L2 and their receptor PD-1 on tumor cells, leading to T-cell exhaustion and attenuation of effector T-cell responses [40]. Additionally, tumor cells escape IFNγ-induced immune elimination by downregulating the expression of signaling molecules, switching to alternative signaling pathways that are associated with tumor promotion and survival, or undergoing G0/G1 cell cycle arrest and entering a quiescent state [38]. Furthermore, some studies indicate that the available concentration of IFNγ might dictate its tumor-supportive or opposing role, with low levels of the cytokine inducing metastasis and tumorsphere formation, while high-dose infusion leads to tumor regression [41].

Moreover, IFNG and 4 other genes, namely, CXCR5, IL2RG, TBX21, and CD40LG, were found to be significantly differentially expressed between the M0 and M2 mammosphere formation groups in an extended cohort of 60 patients. These genes are involved in the regulation of different immune-related processes [41–45] and were downregulated in the M2 group, further supporting our hypothesis. In addition, these genes were evaluated in relation to the clinicopathological data of our cohort of patients as well as in an extended external TCGA cohort. The analysis revealed that increased expression of these genes was correlated with a better prognosis and increased progression-free survival in breast cancer patients. Therefore, although indirect, our data suggest that the presence of CSCs in breast tumors could influence infiltration and immune response activity at the tumor site and impact the prognosis and survival of cancer patients. The implication of the immune context in patient prognosis is well supported by published data, which indicate that low CD8 + T-cell infiltration could be associated with cancer stem cell maintenance and tumor recurrence in pancreatic cancer [38], while in breast cancer, several studies have demonstrated the positive effect of infiltrating CD8 + lymphocytes on patient prognosis and survival [46–50]. This information is particularly relevant in the context of newly emerging immunotherapy approaches for improving breast cancer clinical management [51] and opens new perspectives regarding the interplay between CSCs and the tumor immune microenvironment as an important variable to be addressed when designing therapeutic strategies.

## Conclusion

Collectively, our findings imply a robust correlation between the enrichment of CSCs in primary tumor biopsies and the suppression of immune activity within breast tumors. The only clinicopathological feature associatiated with mammosphere formation was the clinical tumor size, which is not surprising considering that CSCs presence has been solely reported in correlation with tumor recurrence. In light of the absence of clear clinicopathological features that can predict CSCs presence, these observations not only present novel prospects, but also highlight potential challenges in the realm of immunotherapeutic approaches for breast cancer. Nonetheless, conducting thorough investigations through single-cell analysis and spatial transcriptomics holds the potential to yield valuable insights into the activation state of immune cells at the interface with CSCs. Furthermore, such analyses can shed new light on the underlying mechanisms governing their intricate interplay, with important implications for advancing the field of immunotherapy in breast cancer.
